# Secreted factors from dental pulp stem cells improve Sjögren’s syndrome via regulatory T cell-mediated immunosuppression

**DOI:** 10.1186/s13287-021-02236-6

**Published:** 2021-03-16

**Authors:** Mayu Matsumura-Kawashima, Kenichi Ogata, Masafumi Moriyama, Yuka Murakami, Tatsuya Kawado, Seiji Nakamura

**Affiliations:** Section of Oral and Maxillofacial Oncology, Division of Maxillofacial Diagnostic and Surgical Sciences, Faculty of Dental Science, Kyushu University, 3-1-1 Maidashi, Higashi-ku, Fukuoka, 812-8582 Japan

**Keywords:** Dental pulp stem cell, Secreted factor, Sjögren’s syndrome, Regulatory T cell

## Abstract

**Background:**

Sjögren’s syndrome (SS) is a chronic autoimmune disease primarily characterized by inflammation in the salivary and lacrimal glands. Activated T cells contribute to disease pathogenesis by producing proinflammatory cytokines, which leads to a positive feedback loop establishment. The study aimed to evaluate the effects of secreted factors derived from dental pulp stem cells (DPSCs) or bone marrow mesenchymal stem cells (BMMSCs) on hyposalivation in SS and to investigate the mechanism involved.

**Methods:**

Eighty percent confluent stem cells were replenished with serum-free Dulbecco’s modified Eagle’s medium and incubated for 48 h; following which, conditioned media from DPSCs (DPSC-CM) and BMMSCs (BMMSC-CM) were collected. Cytokine array analysis was performed to assess the types of cytokines present in the media. Flow cytometric analysis was performed to evaluate the number of activated T cells cultured in DPSC-CM or BMMSC-CM. Subsequently, DPSC-CM or BMMSC-CM was administered to an SS mouse model. The mice were categorized into the following groups (*n* = 6 each): non-treatment, Dulbecco’s modified Eagle’s medium (−), BMMSC-CM, and DPSC-CM. Histological analysis of the salivary glands was performed. The gene and protein expression levels of cytokines associated with T helper subsets in the submandibular glands (SMGs) were evaluated.

**Results:**

DPSC-CM contained more secreted factors with tissue-regenerating mechanisms, such as cell proliferation, anti-inflammatory effects, and immunomodulatory effects. DPSC-CM was more effective in suppressing the activated T cells than other groups in the flow cytometric analysis. The stimulated salivary flow rate increased in SS mice with DPSC-CM compared with that in the other groups. In addition, the number of inflammation sites in SMGs of the mice administered with DPSC-CM was lower than that in the other groups. The expression levels of *interleukin* (*Il*)*-10* and transforming growth factor-β1 were upregulated in the DPSC-CM group, whereas those of *Il-4* and *Il-17a* were downregulated. The DPSC-CM-administered group presented with a significantly increased percentage of regulatory T (Treg) cells and a significantly decreased percentage of type 17 Th (Th17) cells compared with the other groups.

**Conclusions:**

These results indicated that DPSC-CM ameliorated SS by promoting Treg cell differentiation and inhibiting Th17 cell differentiation in the mouse spleen.

**Supplementary Information:**

The online version contains supplementary material available at 10.1186/s13287-021-02236-6.

## Background

Sjögren’s syndrome (SS) is a chronic, systemic autoimmune disorder characterized by the inflammation of exocrine glands and functional impairment of the salivary and lacrimal glands [1]. T cells (mostly CD4-expressing T helper [Th] cells) form a large part of the lymphocytic infiltrate observed in the salivary and lacrimal gland tissues of SS patients, particularly during the earlier stages of the disease [1, 2]. Furthermore, a remarkable reduction in the numbers of regulatory T (Treg) cells in the salivary glands and CD4^+^CD25^+^ T cells in the peripheral blood have been observed in these patients [2]. The inflammatory tissues in the salivary glands of patients with SS consist predominantly of T cells, particularly, the type 1 Th (Th1) cells [3]; however, type 2 (Th2) and type 17 (Th17) cells have also been observed in the tissues [4], demonstrating the complexity of the pathogenesis of SS. Th1 cells mainly produce interleukin (IL)-2 and interferon gamma (IFN-γ) and are involved in cellular immunity [5]. Th2 cells are mainly responsible for humoral immunity via the activation of B cells and mast cells, and the production of immunoglobulin E; they primarily produce IL-4, IL-5, and IL-13 [6]. IL-2 modulates the expression levels of the receptors of other cytokines and transcription factors during Th cell differentiation, thereby either promoting or inhibiting the cytokine cascades associated with Th cell development state [7]. Thus, IL-2 promotes the differentiation of naïve T cells into Th1 cells. These subsets are then controlled mutually by their cytokines. Furthermore, Th17 cells have been shown to play a crucial role in inducing autoimmunity and allergic inflammation [5]. Several studies have reported that Th17 cell is characterized by the production of IL-17, a cytokine that is not produced by Th1 and Th2 cells [8, 9]. In addition, Th17 cells and their associated cytokines have been implicated in the pathogenesis of SS [10–12]. On the other hand, Treg cells suppress autoreactive lymphocytes via cell–cell contact or the release of soluble mediators, such as IL-10 and transforming growth factor-β1 (TGF-β1) [13].

The treatment of SS is challenging owing to the effect of SS on the immune system. Traditional pharmacological therapies, such as pilocarpine, which is used to stimulate residual acinar cells, are not aimed at the cause of the disease; therefore, they cannot repair the damaged gland and restore its secretory function [14].

Stem cells, such as bone marrow mesenchymal stem cells (BMMSCs) and dental pulp stem cells (DPSCs), have been reported to exert immunomodulatory effects on various activated lymphoid cells, including T cells, B cells, natural killer cells, and dendritic cells [15, 16]. Their low immunogenicity and immunoregulatory potentials offer a promising new treatment for severe refractory autoimmune diseases [17–19]. The therapeutic effects of BMMSC or DPSC infusion have been demonstrated in experimental and clinical SS [20, 21]. However, mesenchymal stem cells (MSCs) have several drawbacks, such as a high capital investment, expensive cell culture, complicated safety, and quality management issues with regard to cell handling, and patient discomfort due to the invasive procedure required for cell collection [22]. Moreover, the implanted MSCs do not survive for long and disappear several weeks after transplantation [23].

It was recently revealed that implanted MSCs secrete a variety of paracrine factors such as growth factors and chemokines that have immunomodulatory effects [23, 24] and can accumulate in conditioned media during cell culture [25]. We previously reported that serum-free conditioned media from MSCs contain numerous cytokines [26–28]. However, studies examining the effects of factors secreted from BMMSCs or DPSCs on inflammatory autoimmune diseases, including SS, are lacking; moreover, the underlying mechanisms involved in these immunomodulatory effects remain unclear.

The treatment of SS is difficult and challenging [14]. In contrast to other inflammatory autoimmune diseases, including rheumatoid arthritis, the blocking of TNF-α had very little effect in patients with SS [3]. The aim of the present study was to evaluate whether secreted factors derived from DPSCs or BMMSCs (DPSCs conditioned media [DPSC-CM] or BMMSCs conditioned media [BMMSC-CM]) exert therapeutic effects in mouse models of SS.

## Methods

### Cell preparation

Three different lots of human DPSCs and BMMSCs were purchased from Lonza, Inc. (Walkersville, MD, USA). DPSCs were cultured in DPSCs basal medium (Lonza, Inc.) containing DPSC SingleQuots (Lonza, Inc.) at 37 °C in 5% CO_2_/95% air. BMMSCs were cultured in MSCs basal medium (Lonza, Inc.) containing MSC-GM SingleQuots (Lonza, Inc.) at 37 °C in 5% CO_2_/95% air. After primary culture, the cells were sub-cultured at a density of approximately 1 × 10^4^ cells/cm^2^. Cells from the third to sixth passages were used for the experiments.

Peripheral blood mononuclear cells (PBMCs) were obtained from six healthy volunteers (two men and four women; age, 61.1 ± 11.6 years) according to the ethics statement.

### Preparation of conditioned media

After achieving 80% confluence, DPSCs or BMMSCs were replenished with serum-free Dulbecco’s modified Eagle’s medium (DMEM [−]; Gibco, Rockville, MD, USA) containing antibiotic–antimycotic solution. The cell-cultured conditioned media were collected after 48 h of incubation. Subsequently, the conditioned media were centrifuged at 440×*g* for 5 min at 4 °C. The supernatant was collected, centrifuged at 17,400×*g* for 3 min at 4 °C, and filtered using 0.22-μm pore filters (Millex®-GP; Merck Millipore Ltd., Billerica, MA, USA). DPSC-CM or BMMSC-CM was stored at − 80 °C before use for the experiments detailed as follows.

### Cytokine antibody array

A cytokine array analysis was performed via laser scanning using 174 human cytokine array plates to assess the types of cytokines present in DPSC-CM and BMMSC-CM (Quantibody® Human Cytokine Array 6000; RayBiotech, Inc., Norcross, GA, USA). Each scan was performed in duplicate, and data were calculated as the ratio of the cytokine level in DPSC-CM to that in BMMSC-CM.

### Isolation and culture of PBMCs

Blood samples (8 mL) from six healthy volunteers were collected in BD Vacutainer® CPT™ (Nippon Becton Dickinson Company, Ltd., Tokyo, Japan). PBMCs were isolated by Ficoll-Paque gradient centrifugation, as described previously [29]. Briefly, immediately following blood collection, the tubes were inverted 10 times and centrifuged in a swinging-bucket rotor at 800×*g* for 30 min at room temperature. After centrifugation, 3 mL of plasma was removed from the uppermost layer. The PBMCs layer was gently suspended in the remaining plasma and transferred to 15 mL conical tubes and washed with phosphate-buffered saline (PBS) by centrifugation at 400×*g* for 10 min. PBMCs were washed with PBS and cultured in RPMI 1640 (Gibco, Rockville, MD, USA) containing an antibiotic–antimycotic solution (100 units/mL penicillin G, 100 mg/mL streptomycin, 0.25 mg/mL amphotericin B; Gibco), and 10% heated-inactivated fetal bovine serum (FBS; Sigma-Aldrich, St. Louis, MO, USA).

### Flow cytometric analysis

The collected PBMCs (5 × 10^5^ cells/mL) were incubated with phytohemagglutinin (PHA, 5 μg/mL) for 4 days to activate T cells. The culture medium was aspirated and gently washed with PBS (3 times). The T cells were incubated for 72 h in the following media: DPSC-CM, BMMSC-CM, and DMEM. Subsequently, they were collected, washed with eBioscience Flow Cytometry Staining Buffer (Thermo Fisher Scientific, Waltham, MA, USA), rinsed, and incubated at normal temperature for 20 min in the dark with APC anti-human CD25 (Miltenyi Biotec, Bergisch, Gladbach, Germany) or APC anti-human CD69 (Miltenyi Biotec). The cells were fixed and permeabilized in Fixation Buffer (BioLegend, San Diego, CA, USA) and Intercellular Staining Perm Wash Buffer (10X) (BioLegend), followed by staining with FITC anti-human CD4 (BioLegend). APC mouse IgG_2A_ (R&D System, Minneapolis, MN, USA), FITC mouse IgG isotype control (Abcam, Cambridge, UK), and mouse IgG_1_ PerCP-conjugated antibody (R&D System) served as negative controls. The BD FACSVerse™ Flow Cytometer (Becton, Dickinson and Company, Franklin Lake, NJ, USA) and BD FACSuite™ software were used to acquire and analyze the FACS data.

### Mice model and injection of DPSC-CM or BMMSC-CM

Nonobese diabetic (NOD) female mice (13 weeks old) purchased from the Charles River Laboratories Japan (Yokohama, Japan) were used as the primary SS model [30, 31]. The mice were categorized into the following groups based on the material performing an intravenous injection twice a week (*n* = 6 per group): (1) non-treatment group, not administered anything; (2) DMEM (−) group (500 μL at a time), DMEM (−) administered; (3) BMMSC-CM group (500 μL at a time), BMMSC-CM administered; and (4) DPSC-CM group (500 μL at a time), DPSC-CM administered. The mice were euthanized at 2 weeks after intravenous injection.

### Measurement of stimulated saliva flow

The NOD mice were anesthetized with chloral hydrate (0.4 g/kg body weight), and the stimulated saliva flow was measured as described previously [21, 32]. At 3 min after pilocarpine intraperitoneal injection (0.05 mg/100 g body weight), a micropipette was used to collect the whole saliva from the oral cavity for 10 min; following which, the amount of saliva collected was calculated.

### Histological analysis

Hematoxylin and eosin (H&E) staining and immunohistochemistry were performed as described previously [26]. Briefly, dissected submandibular glands (SMGs) were fixed in 4% PFA, dehydrated in graded ethanol, cleared in xylene, and embedded in paraffin. The samples were cut to create 5-μm-thick histological sections, stained with H&E, and analyzed under a light microscope.

### Terminal deoxynucleotidyl transferase-mediated dUTP nick-end labeling staining

Terminal deoxynucleotidyl transferase-mediated dUTP nick-end labeling (TUNEL) staining was performed (Click-iT Plus TUNEL Assay with Alexa Fluor 647, Thermo Fisher Scientific Inc., Waltham, MA, USA) to detect apoptotic cells, according to the manufacturer’s instructions. Images of the sections were taken with a fluorescence microscope (BZ-X810, KEYENCE, Osaka, Japan; *n* = 8 per group). The percentage of TUNEL-positive cells per total number of cells was calculated in SMGs of each group.

### Enzyme-linked immunosorbent assay analyses (ELISA)

The concentrations of anti-double-stranded DNA (dsDNA) and anti-SSA in the mice were measured using mouse anti-dsDNA and mouse anti-Ro52/SSA ELISA kits (Signosis Inc., Santa Clara, CA, USA), according to the manufacturer’s protocols. The serum samples were diluted at 1:50. Furthermore, ELISA for Th1/Th2/Th17/Treg-related markers (IL-2, IFN-γ, IL-4, IL-17A, IL-10, and TGF-β1) was performed using a mouse SMG (20 mg per group) according to the manufacturer’s protocol (multi-analyte ELISA kit; MEM-003A; QIAGEN).

### Extraction of RNA and synthesis of complementary DNA

Total RNAs isolated from SMGs (*n* = 6 per group) were dissected with a QIAshredder and RNeasy mini extraction kit (QIAGEN) as described previously [33]. One microgram of total RNA was prepared and used for the synthesis of cDNA. The RNA was incubated for 1 h at 42 °C with 20 units of RNase inhibitor (Promega Japan, Tokyo, Japan), 0.5 μg of Oligo (dT)_12–18_ primer (Thermo Fisher Scientific Inc., Waltham, MA, USA), 0.5 mM deoxyribonucleotide triphosphate (AB0196; Thermo Fisher Scientific Inc., Waltham, MA, USA), 10 mM of dithiothreitol, and 100 units of RNA reverse transcriptase (Life Technologies Japan Ltd., Tokyo, Japan).

### Quantitative reverse transcriptase-polymerase chain reaction

Quantitative reverse transcriptase-polymerase chain reaction (PCR) was used to determine the mRNA levels of the cytokines. The resulting cDNA was amplified using the PowerUp™ SYBR® Green Master Mix (Thermo Fisher Scientific Inc., Waltham, MA, USA) in the AriaMX Real-Time PCR instrument (version 1.7; Agilent Technologies, Inc.). The levels of mRNA for *Il-2*, *Inf-γ*, *Il-10*, *Il-4*, *Il-6*, *Il-17a*, and *Tgf-β1* were analyzed. Target mRNA levels were expressed relative to that for *β-actin* (housekeeping gene). The ∆∆-CT method was applied for the analyses. All analyses were performed in triplicate. The following PCR primers were used for further specific analysis: *Il-2*, 5′-ACTGTTGTAAAACTAAAGGGCTCTG-3′ and 5′-GCAGGAGGTACATAGTTATTGAGGG-3′; *Inf-γ*, 5′-CTTGGCTTTGCAGCTCTTCC-3′ and 5′-CACATCTATGCCACTTGAGTTAAAA-3′; *Il-4*, 5′-TCTTTCTCGAATGTACCAGGAGC-3′ and 5′-TGTGAGGACGTTTGGCACATC-3′; *Il-6*, 5′-AGTTCCTCTCTGCAAGAGACTTC-3′ and 5′-TTTCCACGATTTCCCAGAGAAC-3′; *Il-17a*, 5′-CAGGGAGAGCTTCATCTGTGTCTC-3′ and 5′-TGCGCCAAGGGAGTTAAAGAC-3′; *Il-10*, 5′-GGTAGAAGTGATGCCCCAGG-3′ and 5′-AATCGATGACAGCGCCTCAG-3′; *Tgf-β1*, 5′-CAGGGAGAGCTTCATCTGTGTCTC-3′ and 5′-TGCGCCAAGGGAGTTAAAGAC-3′; and *β-actin*, 5′-CACTCCTAAGAGGAGGATGGTCG-3′ and 5′-CAGACCTGGGCCATTCAGAAA-3′.

### Isolation of lymphocytes from the spleen

Splenic lymphocytes were isolated from the NOD mice as described previously [34]. Briefly, the spleens were dissected and carefully placed in a 60-mm dish containing 3 mL of RPMI 1640 (Gibco) with 2% FBS. A square piece of sterile 70-μm nylon mesh (Corning Incorporated, New York, USA) was placed over the tissue using sterilized forceps. The spleens were gently smashed against the mesh, and almost all of it was suspended in the medium. The suspension was pipetted up and down a few times to break up the remaining soluble clumps. A new square piece of nylon mesh was placed over the opening of a 15-mL conical tube, and the suspension was filtered through to remove the debris. Once the suspension was filtered into the 15-mL tube, the remainder of the tube was filled with RPMI 1640 and inverted a few times. The cells were centrifuged at 300×*g* for 5 min at 4 °C. Then, one volume of the cell suspension was diluted with 10 volumes of 1 × Red Blood Cell Lysis Solution (pluriSelect, San Diego, CA, USA) and vortexed for 5 s. The cells were centrifuged at 300×*g* for 10 min at room temperature. The supernatant was aspirated after centrifugation and resuspended in 10 mL of cold PBS.

For the intracellular and extracellular staining, the lymphocytes (1 × 10^6^) were stained using the PerFix-nc Kit (BEKMAN COULTER, Inc., Brea, CA, USA) according to the manufacturer’s instructions. After staining with FITC anti-mouse CD4 (BioLegend), the lymphocytes were stained with PE anti-CD25 (BioLegend) and PerCP-Cy™5.5 anti-T box gene expressed in T cells (T-bet; BD Biosciences), PE anti-CD25 and APC anti-GATA3 (BioLegend), PE anti-CD25 and APC anti-forkhead box protein P3 (Foxp3; Sigma-Aldrich), or PE anti-CD25 and APC anti-retinoic acid-related orphan receptor γ (RORγ; Miltenyi Biotec). Thereafter, the BD FACSVerse™ Flow Cytometer (Becton, Dickinson and Company, Franklin Lake, NJ, USA) and BD FACSuite™ software were used to acquire and analyze the FACS data.

### Immunohistochemical analysis

Immunohistochemical staining was performed for T-bet (1:500; sc-21763, SANTA CRUZ BIOTECHNOLOGY, INC., Dallas, TX, USA) to evaluate the Th1 cells, GATA3 (1:500; sc-268, SANTA CRUZ BIOTECHNOLOGY, INC., Dallas, TX, USA) to evaluate Th2 cells, Foxp3 (1:200; NB100-39002, Novus Biologicals, Centennial, CO, USA) to evaluate Treg cells, and RORγ (1:1000; ab207082, Abcam, Cambridge, UK) to evaluate Th17 cells. The sections were rehydrated, subjected to antigen retrieval using Dako Target Retrieval Solution (pH 9.0; Dako North America Inc., Carpinteria, CA, USA) for 10 min at 121 °C, blocked for endogenous peroxidase with 0.3% H_2_O_2_ in methanol, and incubated for 30 min. After washing with PBS, the sections were blocked for non-specific binding using Blocking One Histo (Nakalai Tesque, Inc., Kyoto, Japan) for 15 min at room temperature and then incubated with the primary antibody overnight at 4 °C. Subsequently, the sections were reacted with Peroxidase Stain DAB Kit (Nakalai Tesque, Inc., Kyoto, Japan) for 1 h and developed with 3,3′-diaminobenzidine (DAB) solution. Hematoxylin counterstaining was performed following the DAB reaction.

### Immunoblotting

Mouse spleen was lysed in T-PER™ Tissue Protein Extraction Reagent (Thermo Fisher Scientific). Equal amounts of protein were analyzed using SDS-PAGE followed by electrophoretic transfer to polyvinylidene difluoride (PVDF) membranes (Millipore Ltd.), which were blocked for 1 h with blocking buffer (5% skim milk in TBS) and incubated overnight at 4 °C with a primary antibody. Antibodies used for immunoblotting were as follows: mouse polyclonal anti-nuclear factor of activated T cells (NFAT) (Abcam), rabbit polyclonal anti-nuclear factor kappa-light-chain-enhancer of activated B cells (NF-κB) (Abcam), rabbit monoclonal anti-Foxp3 (Abcam), rabbit polyclonal anti-phospholylation-Smad2/3 (p-Smad2/3) (Cell Signaling Technology, Kyoto, Japan), rabbit polyclonal anti-Smad2/3 (Cell Signaling Technology), rabbit polyclonal anti-extracellular signal-regulated kinase (ERK) 1/2 (Cell Signaling Technology), anti-phosphorylation-ERK (p-ERK) 1/2 (Cell Signaling Technology), and rabbit monoclonal anti-β-actin (Cell Signaling Technology).

### Statistical analyses

All experiments were conducted in triplicate and repeated at least twice. Group means and standard deviations were calculated for each measured parameter. Statistical differences were evaluated using Student’s *t* test, Mann–Whitney *U* test, and Tukey’s honest significant difference test. A *p* value of < 0.05 was considered statistically significant, and a *p* value of < 0.01 was considered highly significant.

## Results

### DPSC-CM and BMMSC-CM analyses

**Fig. 1:**
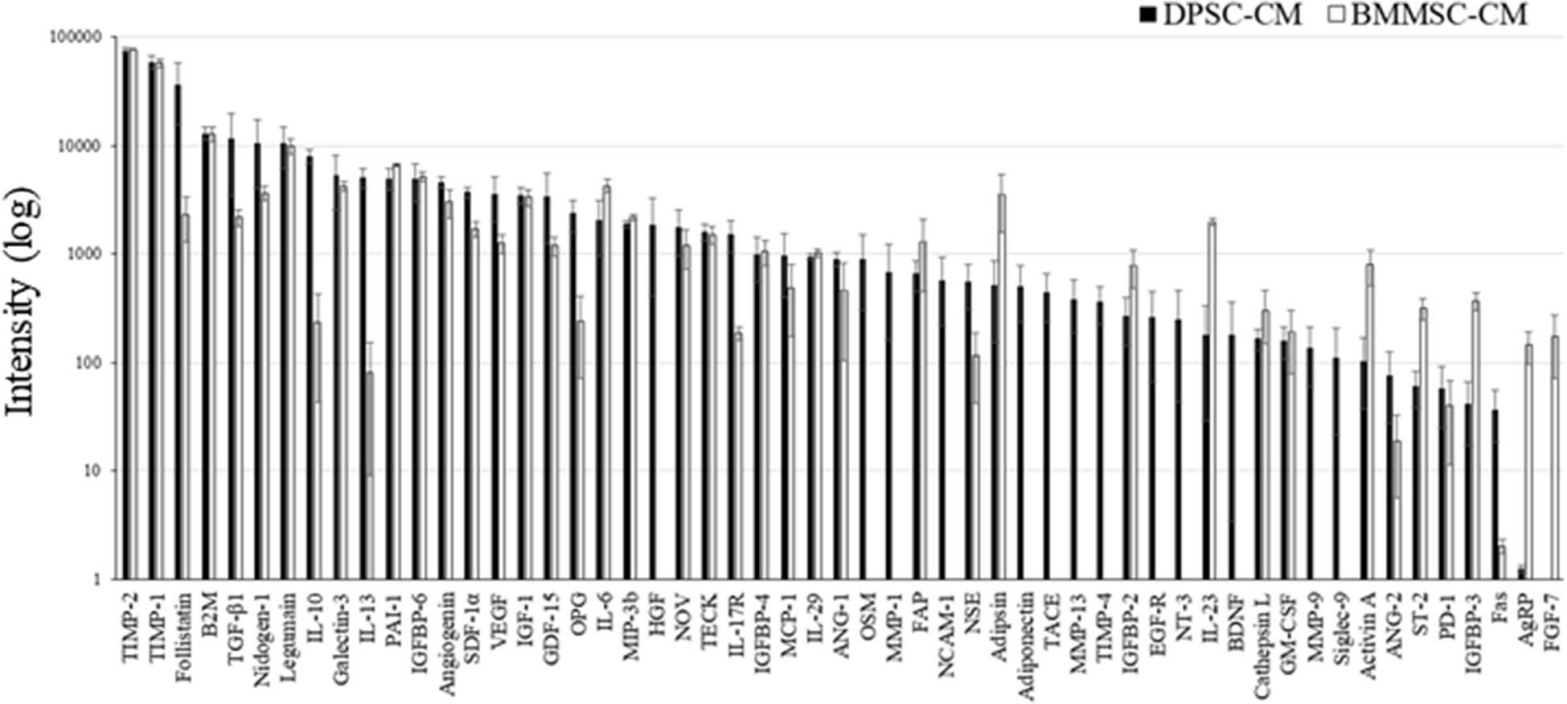
DPSC-CM contains more anti-inflammatory factors and immunomodulatory factors than BMMSC-CM. Graph showing the factors expressed in DPSC-CM and BMSC-CM (*n* = 3 each). The *y*-axis indicates the relative intensity

After performing the cytokine array (Quantibody® Human Cytokine Array 6000; RayBiotech), which compared DPSC-CM with BMMSC-CM, 55 representative growth factors, anti-inflammatory cytokines, and tissue regeneration factors were detected (Fig. 1). DPSC-CM contained more anti-inflammatory cytokines than BMMSC-CM (IL-10, 34 times; IL-13, 63 times; and follistatin, 15 times) (Table 1). Furthermore, hepatocyte growth factor (HGF), sialic acid-binding Ig-like lectin 9, neural cell adhesion molecule-1, neurotrophin-3, and brain-derived neurotrophic factor were detected only in DPSC-CM (Table 1).

**Table 1:** Classification of DPSC-CM factors vs. MSC-CM factors

	Anti-inflammatory factors (Intensity)	
	DPSC-CM	BMMSC-CM
TGF-β1	11,623	2186
IL-10	7989	234
IL-13	5098	80
IGF-1	3521	3324
TECK	1609	1513
IL-29	943	1019
Adiponectin	502	0
Siglec-9	396	0
GM-CSF	159	188
	Nerve regeneration-related factors (intensity)	
	DPSC-CM	BMMSC-CM
HGF	1857	0
NCAM-1	573	0
NSE	556	150
NT-3	250	0
BDNF	179	0
	Anti-fibrotic factors (intensity)	
	DPSC-CM	BMMSC-CM
Follistatin	36,241	2328
HGF	1857	0
	Angiogenesis-related factors (intensity)	
	DPSC-CM	BMMSC-CM
Angiogenin	4622	3025
VEGF	3549	1249
ANG-1	895	459

*TIMP-2* tissue inhibitor of metalloproteinase-2, *TIMP-1* tissue inhibitor of metalloproteinase-1, *B2M* β-2 microglobulin, *TGF-β1* transforming growth factor-β1, *IL-10* interleukin-10, *IL-13* interleukin-13, *PAI-1* plasminogen activator inhibitor-1, *IGFBP-6* insulin-like growth factor-binding protein-6, *SDF-1α* stromal cell-derived factor 1α, *VEGF* vascular endothelial growth factor, *IGF-1* insulin-like growth factor-1, *GDF-15* growth and differentiation factor 15, *OPG* osteoprotegerin, *IL-6* interleukin-6, *MIP-3b* macrophage inflammatory protein-3β, *HGF* hepatocyte growth factor, *TECK* thymus-expressed chemokine, *IL-17R* interleukin-17 receptor, *IGFBP-4* insulin-like growth factor-binding protein-4, *IL-29* interleukin-29, *ANG-1* angiopoietin-1, *OSM* oncostatin M, *MMP-1* matrix metalloproteinase-1, *FAP* fibroblast activation protein, *NCAM-1* neural cell adhesion molecule-1, *NSE* neuron-specific enolase, *TACE* tumor necrosis factor-α convertase, *MMP-13* matrix metalloproteinase-13, *TIMP-4* tissue inhibitor of metalloproteinase-4, *IGFBP-2* insulin-like growth factor-binding protein-2, *EGF-R* epidermal growth factor receptor, *NT-3* neurotrophin-3, *IL-23* interleukin-23, *BDNF* brain-derived neurotrophic factor, *GM-CSF* granulocyte macrophage colony-stimulating factor, *MMP-9* matrix metalloproteinase-9, *Siglec-9* sialic acid-binding immunoglobulin-type lectin-9, *ANG-2* angiopoietin-2, *PD-1* programmed cell death-1, *IGFBP-3* insulin-like growth factor-binding protein-3, *AgRP* agouti-related protein, *FGF-7* fibroblast growth factor-7

### Flow cytometric analysis for activated T cells

The presence of activated T cells (CD3^+^CD25^+^ or CD3^+^CD69^+^ cells) and Th cells (CD4^+^CD25^+^ or CD4^+^CD69^+^ cells) was investigated as described previously [35, 36]. Moreover, RORγ-transgenic mice, an established SS-like sialadenitis mice model, were reported to induce the expression of IL-2-mediated CD25 and CD69 on CD4^+^ T cells [37].

**Fig. 2:**
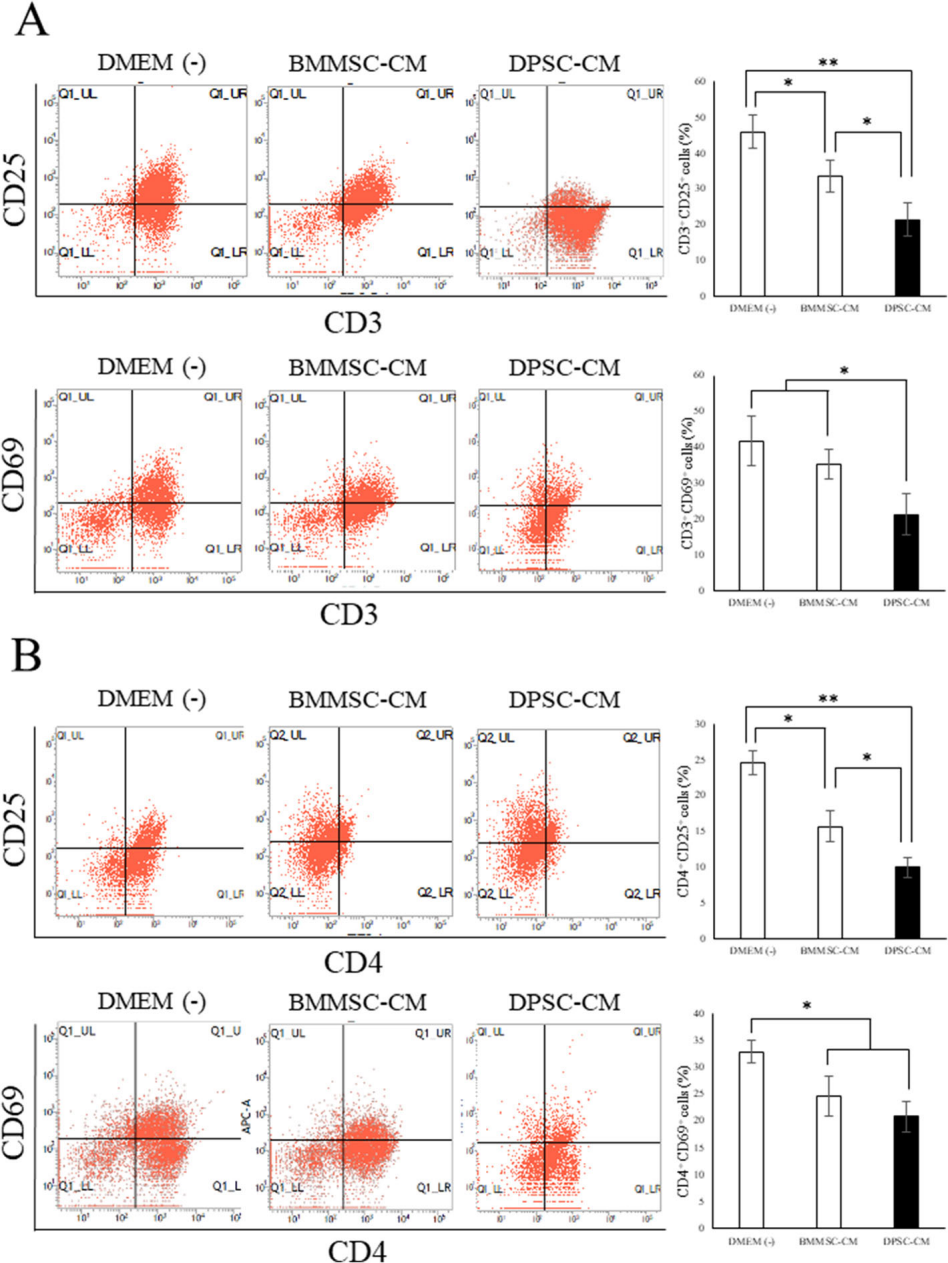
Addition of DPSC-CM regulates the activated T cells. Graphs showing the proportions of activated T cells in the form of CD3^+^CD25^+^ cells or CD3^+^CD69^+^ cells (**a**) and Th cells in the form of CD4^+^CD25^+^ cells or CD4^+^CD69^+^ cells (**b**) were detected. Data are represented as mean ± standard deviation. *n* = 6. **p < 0.01; *p < 0.05

In the present study, flow cytometric analysis suggested that the addition of DPSC-CM could decrease the proportion of CD3^+^CD25^+^ or CD3^+^CD69^+^ cells (Fig. 2a) and CD4^+^CD25^+^ or CD4^+^CD69^+^ cells (Fig. 2b) and that DPSC-CM was more effective in suppressing T cell activation than BMMSC-CM. On the contrary, no changes were observed in the proportion of CD8^+^CD25^+^, CD8^+^CD69^+^, or CD19^+^CD25^+^ (activated B cells) cells, as observed in all the groups (Supplementary Figure 1).

### DPSC-CM alleviates the decrease in the secretion of saliva and inhibits the increase in inflammation in SMGs

NOD mice are used as a primary SS model, which uniquely exhibits salivary and lacrimal gland dysfunction concomitant with the appearance of leukocyte infiltrations in the exocrine glands and the many congenic strains with known genetic differences [38–40].

**Fig. 3:**
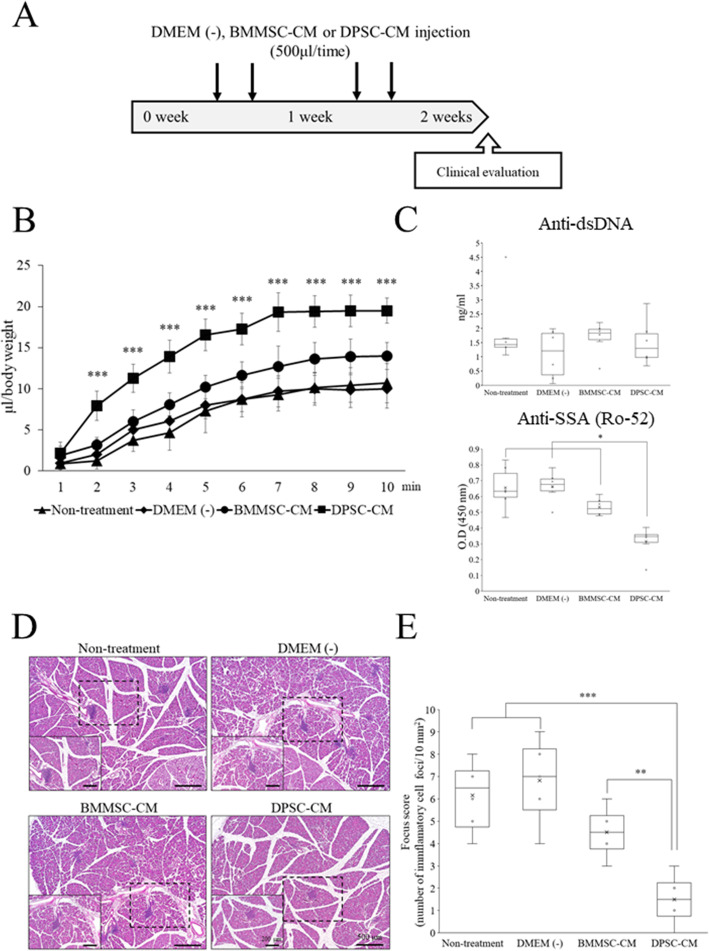
Evaluation of inflammatory infiltration in SMGs of NOD mice. **a** Outline of the experimental protocol. **b** The salivary flow rate in each group at 15 weeks of age. *n* = 6 per group. ***p < 0.001. **c** Quantification of anti-dsDNA (top) and anti-SSA/Ro-52 (bottom) antibodies in the NOD mice at 15 weeks of age. Data represent the mean ± standard deviation. *n* = 6. *p < 0.05. **d** Representative histological images of the submandibular glands for H&E staining in 15-week-old NOD mice. The number of inflammatory cell foci in the DPSC-CM group was lower than that in the other groups. Higher magnifications are displayed in the lower left of each H&E-stained image. Lower magnification bars are 500 μm, while higher magnification bars are 200 μm. **e** The degree of inflammatory infiltration in the submandibular gland was evaluated using the focus score. Data are representative of the mean ± standard deviation. *n* = 6. ***p < 0.001; **p < 0.01

The stimulated saliva flow rate was increased in the 15-week-old mice injected with DPSC-CM when compared with that in the other groups (Fig. 3b). On the other hand, the salivary secretion was slightly increased in the BMMSC-CM group compared with that in the non-treatment or DMEM (−) group (Fig. 3b). Importantly, anti-nuclear antibodies, such as anti-Ro52/SSA antibodies, which are detected in approximately 60% cases of SS [41], were highly detected in serum collected from the non-treatment, DMEM (−), or BMMSC-CM-administered mice when compared with those in the DPSC-CM group; anti-dsDNA did not have a change for each group (Fig. 3c). These results suggest that DPSC-CM injection was effective in alleviating the decrease in fluid secretion and maintaining normal secretory function.

As shown in Fig. 3d, DPSC-CM alleviated inflammation in SMGs of the mice. The focus scores were decreased in the DPSC-CM group compared with those in the non-treatment, DMEM (−), and BMMSC-CM groups, indicating that DPSC-CM alleviated the inflammation of SMGs. Consequently, after 2 weeks, we assessed the safety of BMMSC-CM and DPSC-CM injections. Major organs, such as the brain, lungs, heart, liver, spleen, kidneys, and bladder, had no tumors at least 2 weeks after injection (Supplementary Figure 2A). Moreover, we assessed the mouse kidney and pancreas tissues by H&E staining (Supplementary Figure 2B). Renal disorders, such as nephritis with glomerular basal membrane defects and mesangial cell overgrowth, were not observed in the DPSC-CM group in contrast with the other groups. Pancreatic islet inflammation was also not observed in the DPSC-CM group unlike the other groups.

### mRNA expression levels of cytokines are associated with Th subsets in SMGs

**Fig. 4:**
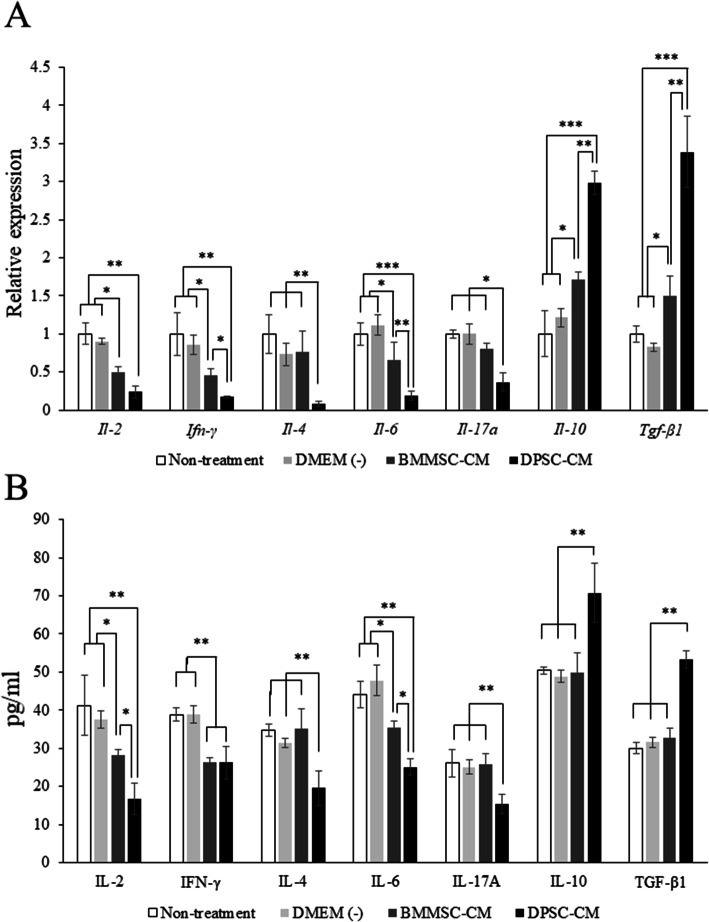
mRNA expression and protein levels of cytokines associated with Th subsets in SMGs. **a** Quantitative RT-PCR analyses of *Il-2*, *Ifn-γ*, *Il-4*, *Il-6*, *Il-17a*, *Il-10*, and *Tgf-β1* in SMGs. *n* = 6 per group. ***p < 0.001; **p < 0.01; *p < 0.05. **b** Amount of Th subset markers in SMGs of the 15-week-old NOD mice. *n* = 6 per group. **p < 0.01; *p < 0.05

The relative mRNA expression levels of the Th1-associated subsets markers, *Il-2* and *Ifn-γ*, were significantly downregulated in SMGs of the DPSC-CM-administered group compared with those in the other groups (Fig. 4a). Similarly, the expression levels of *Il-4* and *Il-17a*, which are associated with the Th2 and Th17 subsets, were significantly downregulated in the DPSC-CM group (Fig. 4a). The expression level of Il-6 was significantly downregulated in the DPSC-CM group (Fig. 4a). On the other hand, *Il-10* and *Tgf-β1* expression levels were significantly upregulated in the DPSC-CM group compared with those in the other groups (Fig. 4a). Similar results were obtained at the protein level (Fig. 4b). These results suggest that DPSC-CM readily induces differentiation into Treg cells compared with the other treatments.

### DPSC-CM decreases apoptosis in SMGs

**Fig. 5:**
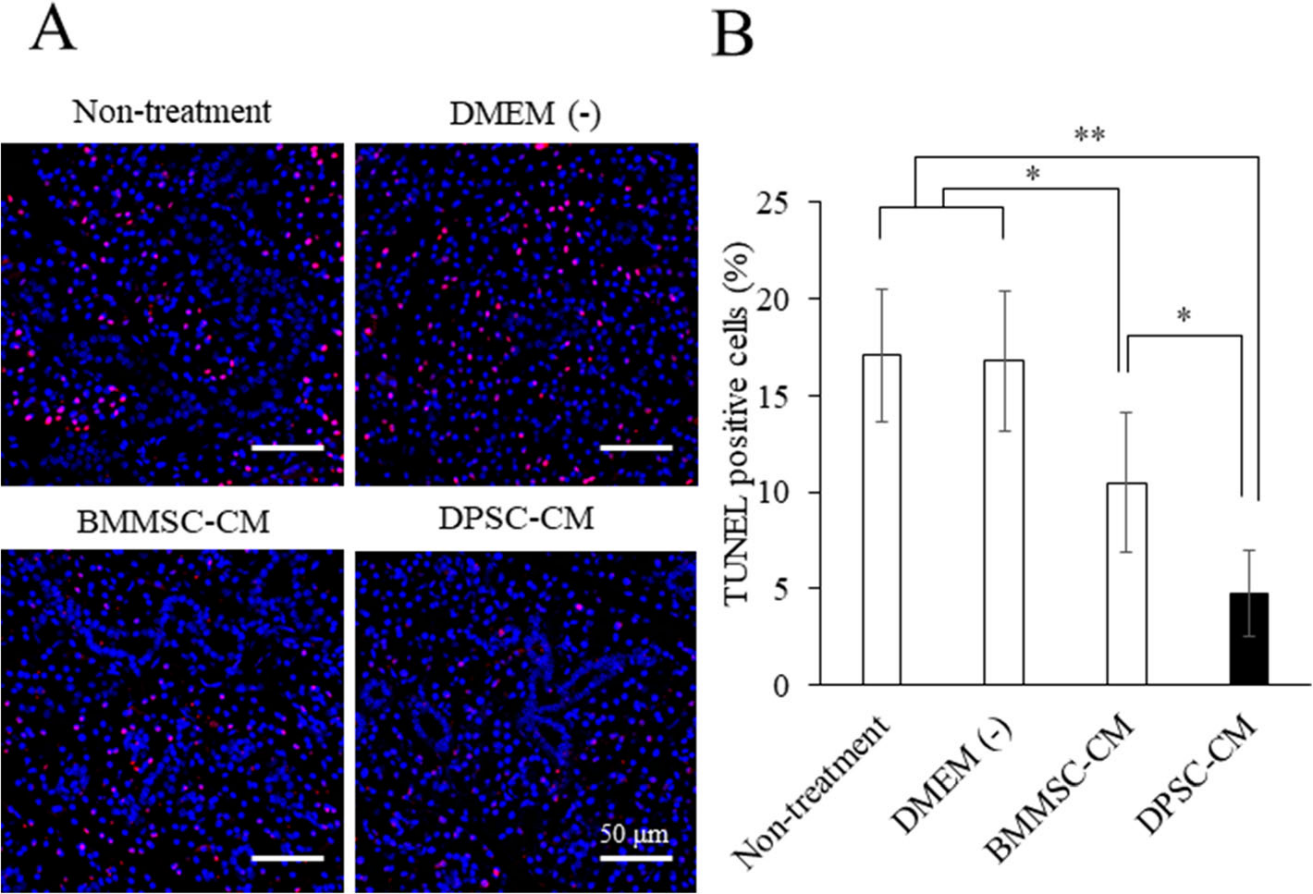
DPSC-CM decreases apoptosis in SMGs. **a** TUNEL assays in SMGs of the NOD mice reveal that the nuclei were stained with DAPI (blue). Bars = 50 μm. **b** Percentage of TUNEL-positive cells per total number of cells in SMGs. Data are representative of the mean ± standard deviation. *n* = 6. **p < 0.01; *p < 0.05

IFN-γ secreted by infiltrating lymphocytes induces ductal apoptosis in sialoadenitis associated with SS, which are responsible for the impairment of gland secretory function [42]. As shown in Fig. 4b, IFN-γ was elevated in both the non-treatment and DMEM (−) groups. Therefore, we investigated whether the numbers of apoptotic cells were increased in SMGs of the NOD mice. The numbers of apoptotic cells were increased in the non-treatment and DMEM (−) groups when compared with those in the BMMSC-CM and DPSC-CM groups (Fig. 5ab). Furthermore, the number of apoptotic cells was significantly decreased in the DPSC-CM-administered group compared with that in the BMMSC-CM-administered group (Fig. 5ab). These results suggest that DPSC-CM has powerful anti-apoptotic effects in SMGs.

### DPSC-CM favors Tregs while suppressing the Th1 and Th17 responses

Next, we investigated whether and how DPSC-CM directly induced the Th subset in the mouse spleen tissue. First, we isolated spleen lymphocytes from the NOD mice. We focused on CD4^+^ T cells in the spleen because DPSC-CM specifically decreased the population of CD4^+^CD25^+^ T cells in the flow cytometric analysis of PBMCs (Fig. 1b).

**Fig. 6:**
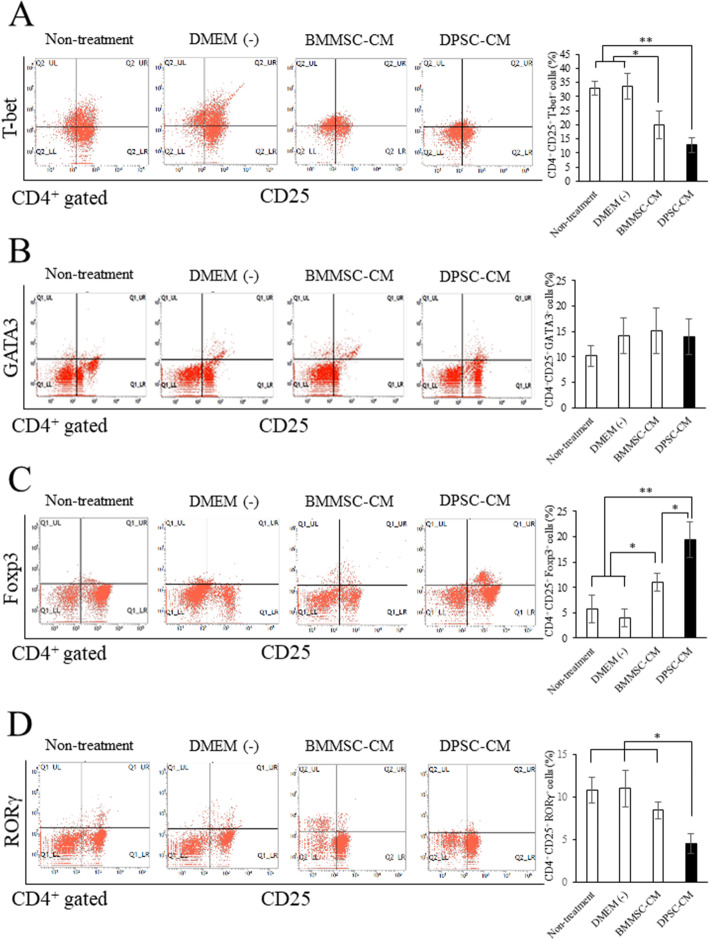
DPSC-CM directly induced the Treg cells in the splenic lymphocytes of the mice. The proportion of CD4^+^CD25^+^T-bet^+^ (Th1) cells (**a**), CD4^+^CD25^+^GATA3^+^ (Th2) cells (**b**), CD4^+^CD25^+^Foxp3^+^ (Treg) cells (**c**), and CD4^+^CD25^+^RORγ^+^ (Th17) cells (**d**) in NOD mice spleen. Each graph on the right side shows the percentage of each T cell subsets. The data represent the mean ± standard deviation. *n* = 6. **p < 0.01; *p < 0.05

**Fig. 7:**
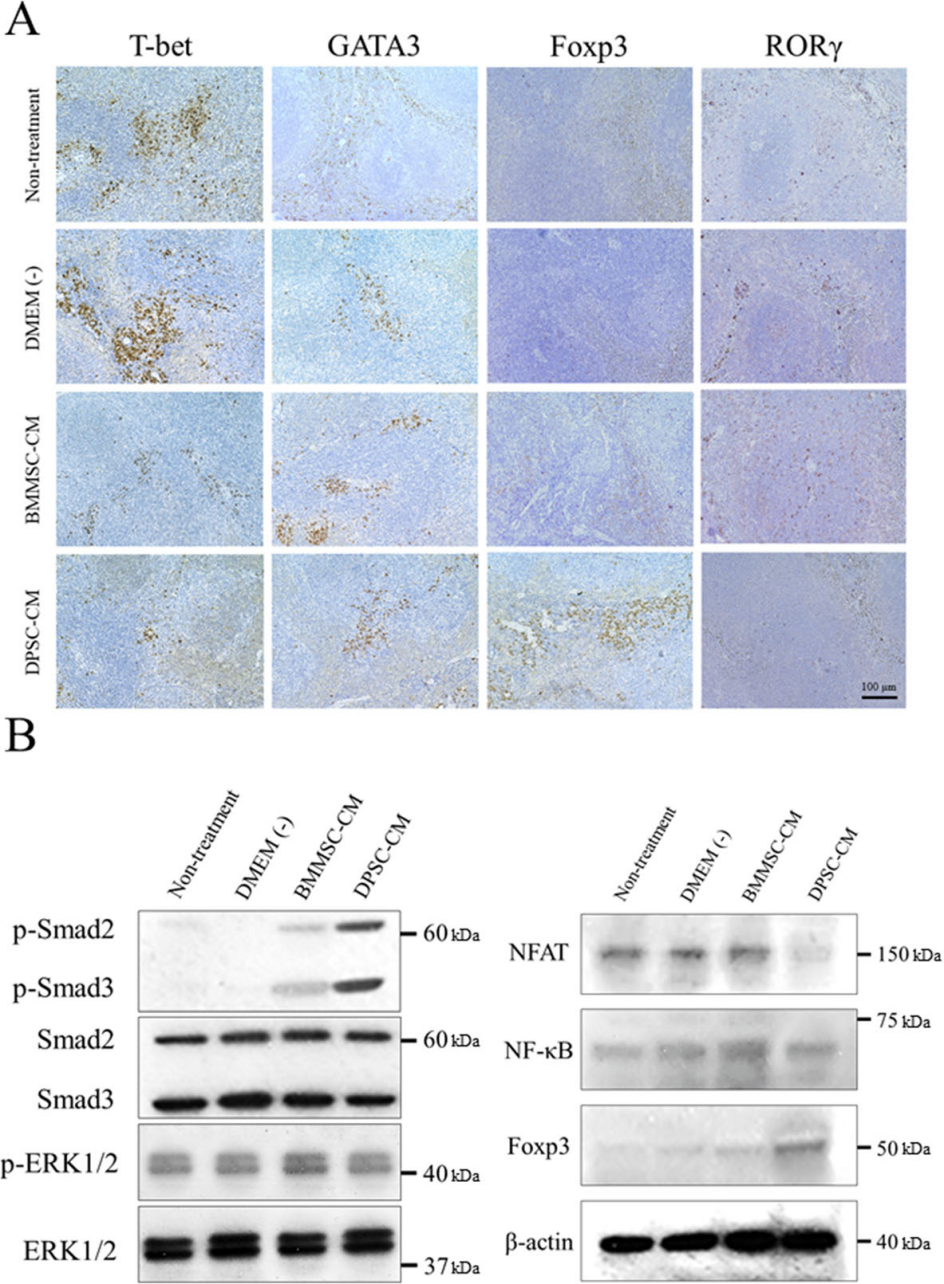
Location of each Th subset in the spleens of NOD mice and immunoblots of the indicated molecules. **a** Immunohistochemical staining for T-bet (Th1 cells), GATA3 (Th2 cells), Foxp3 (Treg cells), and RORγ (Th17 cells). The panels on the upper side show the higher magnifications (bar = 100 μm.) of the spleens. **b** The detection of NFAT, NF-κB, Foxp3, p-Smad2/3, Smad2/3, p-ERK, and ERK protein expression levels was conducted using western blot analysis. β-actin was used as the internal control

The CD4^+^ T cell proportions were not changed in the mice in each group (data not shown). The Th1 (CD4^+^CD25^+^T-bet^+^) cell proportion was increased in the splenic lymphocytes of the animals in the non-treatment and DMEM (−) groups (Fig. 6a). Furthermore, the percentage of Th1 cells was significantly decreased in the DPSC-CM group when compared with that in the other groups (*p* < 0.01, Fig. 6a). However, the proportion of Th2 cells (CD4^+^CD25^+^GATA3^+^) was not altered in the groups (Fig. 6b). The percentage of Treg cells (CD4^+^CD25^+^Foxp3^+^) was significantly increased in the DPSC-CM group (Fig. 6c), whereas that of Th17 cells (CD4^+^CD25^+^RORγ^+^) was significantly decreased when compared with that in the other groups (Fig. 6d). In addition, we investigated the proportion of B cells in mouse spleens. The proportion of regulatory B (Breg) cells (CD19^+^IL-10^+^) was not altered in the groups, while the proportion of plasma cells (CD20^+^CD138^+^) was significantly decreased in the DPSC-CM group (Supplementary Figure 2). Similar results were obtained from the H&E staining of Th subsets in the spleen (Fig. 7a). Taken together, these results suggested that DPSC-CM induces Treg cell differentiation and suppresses Th1 and Th17 cells in splenic lymphocytes.

### DPSC-CM downregulates NFAT and regulates the TGF-β/Smad pathway in the spleen

We finally performed immunoblotting in mouse spleens to understand the molecular mechanisms that mediate changes in the proportion of T cell types. As shown in Fig. 7b, NFAT expression was decreased in the spleens of animals in the DPSC-CM group compared with those in other groups. However, Foxp3 expression was increased in the spleens of animals in the DPSC-CM group compared with those in other groups. The upstream molecule p-Smad2/3 was significantly increased after DPSC-CM treatment, while ERK1/2 and p-ERK1/2 expression did not differ among the groups. These results indicate that NFAT inhibition barely impairs Treg activity and that the primary working mechanism of DPSC-CM treatment was the Treg cell induction through the TGF-β/Smad pathway in mouse spleens.

## Discussion

In this study, we evaluated the therapeutic effects of secreted factors derived from DPSC-CM or BMMSC-CM in a mouse model of SS. DPSC-CM presented with numerous immunosuppressive factors (e.g., TGF-β1, IL-10, and IL-13) compared with BMMSC-CM. Furthermore, quantitative analyses of the cytokines indicated that expression levels of TGF-β1, HGF, IL-10, and IL-13 were significantly higher in DPSC-CM than those in BMMSC-CM (Fig. 1).

NOD mice, the most commonly used animal model of SS, consist of a chronic lymphocytic infiltration in the endocrine and exocrine glands [30, 31]. Severe inflammatory lesions appear in mice at 12–16 weeks of age (the early stage of the clinical phase). A previous study reported that inflammatory lesions appear in 7-week-old mice and have been observed in 14-week-old mice [21]. Moreover, the salivary flow rate declines by 14 weeks in NOD mice [43]. Therefore, we used 14-week-old NOD mice in the current study.

The therapeutic effects of MSCs on tissue engineering and regenerative medicine are attributable, in part, to the paracrine pathways [23, 24] triggered by several factors secreted into the culture media [44]. In a previous study, we reported that BMMSC-CM contains many cytokines, such as the vascular endothelial growth factor, monocyte chemoattractant protein (MCP)-1, MCP-3, and HGF. Recently, many types of biomaterials and stem cell transplantation therapies have been proposed to enhance the anti-inflammatory effects and functional recovery [18, 21]. However, transplanted stem cells exhibit poor differentiation and survival [23]. Furthermore, it has been established that stem cells secrete a variety of growth factors and cytokines [25, 45–47]. The paracrine effects of growth factors and cytokines secreted from implanted stem cells may have anti-inflammatory effects [25, 45–47]. In addition, the paracrine factors secreted by stem cells can accumulate in conditioned media during cell culture [25, 45–47]. Serum-free conditioned media from stem cells have been reported to serve multiple positive functions [25, 45–47]. Yamada et al. reported that the cytokine profiles of stem cells derived from the dental pulp and bone marrow are different; hence, they may have characteristics that are specific to the cells [48]. DPSC-CM has immunoregulatory properties that contribute to tissue repair compared with BMMSC-CM. TGF-β1 plays a critical role in the generation of Th17 cells and immunosuppressive function, thereby contributing to the induction of Treg cells [49]. HGF was originally identified as a potent mitogen for mature hepatocytes. It stimulates the proliferation and proteoglycan synthesis of some mesenchymal cells. Furthermore, it has been indicated that HGF can stimulate the proliferation and differentiation of progenitor cells [50]. IL-13, a known immunosuppressive cytokine, increased IL-10 production in T cells, which attenuated the expression of IL-17A in vitro [51]. It has been reported that TGF-β1 and HGF play important roles in the inhibitory effect of stem cells [48], and TGF-β1 and HGF have been shown to regulate the suppression of SMG inflammation [52]. In the current study, because DPSC-CM contained more secreted factors associated with tissue-regenerating properties, including cell proliferation, anti-inflammatory effects, and immunomodulatory effects (e.g., TGF-β1, IL-10, IL-13, HGF) (Fig. 1), it was speculated that DPSC-CM was more effective than BMMSC-CM in SS.

MSCs have been shown to suppress the immune response by inhibiting T cell proliferation and activation in a mitogen- or allergen-stimulated culture system [53]. In the present study, DPSC-CM strongly inhibited the activation of the CD3^+^CD25^+^T, CD3^+^CD69^+^T, CD4^+^CD25^+^T, and CD3^+^CD69^+^T cells compared with BMMSC-CM (Fig. 2). In addition, the decrease in IL-4, IL-17A, and IFN-γ levels and the increase in IL-10 and TGF-β1 levels in SMGs suggested that DPSC-CM could rectify the immune imbalance in the SS mice model. Furthermore, IFN-γ is secreted mainly by cytotoxic or Th1 T cells and natural killer cells [54]. Zhang et al. reported that desiccating stress and exogenous administration of IFN-γ with desiccating stress exposure increased epithelial apoptosis, indicating that IFN-γ promotes epithelial apoptosis through the extrinsic apoptosis pathway in SS [55]. In the present study, DPSC-CM decreased the expression levels of *Ifn-γ*, *Il-6*, and *Il-17a* and increased those of *Il-10* and *Tgf-β1* in SMGs (Fig. 4). In addition, DPSC-CM injection controlled the apoptosis of ductal cells (Fig. 5).

CD4^+^ T cells are the predominant cells that infiltrate the salivary glands affected by SS [56]. Both Th1 and Th2 cytokine levels are increased in the salivary glands of patients with SS [57, 58]. By contrast, the Th17/Treg ratio in patients with SS was reported to be higher than that in healthy controls but was still lower than that in patients in the experimental group, which indicates a low level of imbalance and an abnormality in the initial differentiation of T cells in the body [59]. This might be due to increases in levels of the specific transcription factor Foxp3 in Treg cells, which inhibits RORγ secretion [60] and leads to a decline in the number of Th17 cells. Th17 cells can secrete the proinflammatory factor IL-17 during immune cell development. Treg cells can also exert an immunoregulatory effect by releasing IL-10 and TGF-β1 to suppress the inflammatory immune response [61]. TGF-β1 induces the differentiation of initial T cells to Treg cells, whereas the combined actions of TGT-β1 and IL-6 induce the differentiation of initial T cells into Th17 cells [62]. In the present study, DPSC-CM induced the differentiation of T cells into Treg cells and suppressed the numbers of Th1 and Th17 cells in the spleen (Figs. 6 and 7a).

We finally investigated the molecular pathways that facilitated Treg cell differentiation in mouse spleens. Several transcription factors, including NFAT and NF-κB, have been identified as interaction partners of Foxp3 [63, 64]. Of note, these transcription factors have also been reported to regulate Foxp3 expression. As shown in Fig. 7b, NFAT expression was decreased in mouse spleens, while NF-κB expression was either unchanged or was slightly decreased in the DPSC-CM group compared with the other groups. Vaeth et al. reported that once T cells differentiate into Treg cells, in vitro and in vivo, they can exert their suppressor functions when the NFAT levels are severely reduced [65]. In accordance, Foxp3^+^ Treg cells express less NFAT and activate NFAT to a lesser extent [66]. These results are in agreement with our results. Moreover, to investigate upstream molecules, we performed immunoblotting for Smad2/3 and ERK1/2, which are believed to be responsible for the Treg cell generation. The results showed that p-Smad2/3 expression was significantly increased in the DPSC-CM group, while ERK1/2 and p-ERK1/2 expression did not differ among the groups (Fig. 7b). Taken together, our data suggest that DPSC-CM participated in the TGF-β/Smad pathway and that cells readily differentiated into Treg cells when the TGF-β/Smad pathway was activated. However, further research is needed to understand the detailed mechanism of this interaction because various pathways participate in Treg differentiation.

## Conclusions

DPSC-CM exerts a protective effect on the secretory function of SMGs. In addition, DPSC-CM alleviates hyposalivation due to SS by decreasing the inflammatory cytokine expression, inducing Tregs in the spleen via the TGF-β/Smad pathway, regulating the local inflammatory microenvironment, and decreasing apoptosis in SMGs. Regulation of the differentiation of T cells by DPSC-CM might be responsible for its immunomodulatory effects. Therefore, this study reveals a new effect of DPSC-CM and provides a therapeutic strategy for SS.

## Supplementary Information

**Additional file 1:** **Supplementary Figure 1.** Flow cytometric analysis for CD8^+^CD25^+^, CD8^+^CD69^+^, or CD19^+^CD25^+^cells. No significant differences were observed between the DMEM (−), BMMSC-CM, and DPSC-CM groups. n.s.: not significant.

**Additional file 2:** **Supplementary Figure 2.** Evaluation of the safety of BMMSC-CM and DPSC-CM. (A) Gross images of BMMSC-CM and DPSC-CM injection twice a week for 2 weeks in 15-week-old NOD mice. (B) Representative H&E-stained histological images of the kidney and pancreas of 15-week-old NOD mice. Bars = 50 μm.

**Additional file 3:** **Supplementary Figure 3.** Flow cytometric analysis of CD19^+^IL-10^+^ and CD20^+^CD138^+^ cells. Each graph on the right side shows the percentages of each B cell subset. The data represent the mean ± standard deviation. *n* = 6. *p < 0.05.

## Data Availability

The datasets used and/or analyzed during the current study are available from the corresponding author on reasonable request.

## Abbreviations

BMMSCs: Bone marrow mesenchymal stem cells
DMEM: Dulbecco’s modified Eagle’s medium
DPSCs: Dental pulp stem cells
ELISA: Enzyme-linked immunosorbent assay
FBS: Fetal bovine serum
H&E: Hematoxylin and eosin
HGF: Hepatocyte growth factor
MSCs: Mesenchymal stem cells
NOD: Nonobese diabetic
PBMC: Peripheral blood mononuclear cells
PBS: Phosphate-buffered saline
PCR: Polymerase chain reaction
