# Supplementary material for: Secreted factors from dental pulp stem cells improve Sjögren’s syndrome via regulatory T cell-mediated immunosuppression
Source: Stem Cell Res Ther. 2021 Mar 16;12:182. doi: 10.1186/s13287-021-02236-6 (PMC7962357; doi:10.1186/s13287-021-02236-6)

**Supplementary information**

**Methods**

*Flow cytometric analysis*

PBMCs were collected and stained as described for the flow cytometric analysis using PerCP-Vio700 anti-human CD8 (Miltenyi Biotec) and PerCP anti-human CD19 (BioLegend). The BD FACSVerse™ Flow Cytometer (Becton, Dickinson and Company, Franklin Lake, NJ, USA) and BD FACSuite™ software were used to acquire and analyze the FACS data.

**Supplementary Figure 1.** Flow cytometric analysis for CD8^+^CD25^+^, CD8^+^CD69^+^, or CD19^+^CD25^+^cells.

No significant differences were observed between the DMEM (−), BMMSC-CM, and DPSC-CM groups. n.s.: not significant.


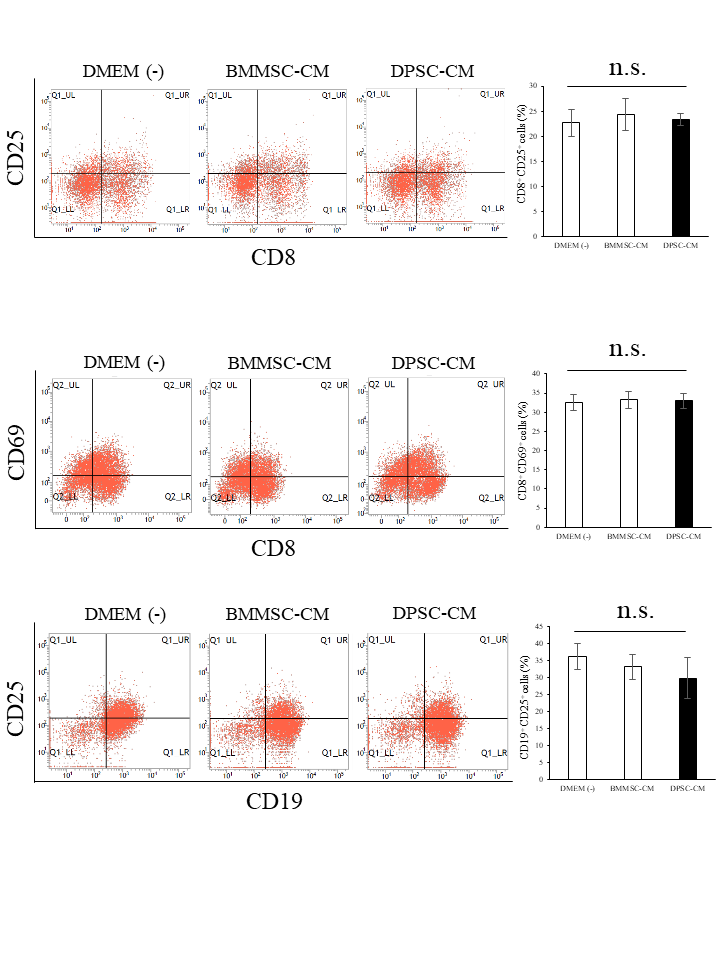

Supplement: Supplementary file 1 — Additional file 1: Supplementary Figure 1. Flow cytometric analysis for CD8+CD25+, CD8+CD69+, or CD19+CD25+cells. No significant differences were observed between the DMEM (−), BMMSC-CM, and DPSC-CM groups. n.s.: not significant. [file 13287_2021_2236_MOESM1_ESM.docx]
