# Supplementary material for: Secreted factors from dental pulp stem cells improve Sjögren’s syndrome via regulatory T cell-mediated immunosuppression
Source: Stem Cell Res Ther. 2021 Mar 16;12:182. doi: 10.1186/s13287-021-02236-6 (PMC7962357; doi:10.1186/s13287-021-02236-6)
Supplement: Supplementary file 2 — Additional file 2: Supplementary Figure 2. Evaluation of the safety of BMMSC-CM and DPSC-CM. (A) Gross images of BMMSC-CM and DPSC-CM injection twice a week for 2 weeks in 15-week-old NOD mice. (B) Representative H&E-stained histological images of the kidney and pancreas of 15-week-old NOD mice. Bars = 50 μm. [file 13287_2021_2236_MOESM2_ESM.docx]

**Supplementary information**

**Methods**

*Hematoxylin and eosin (H&E) staining of kidney and pancreas*


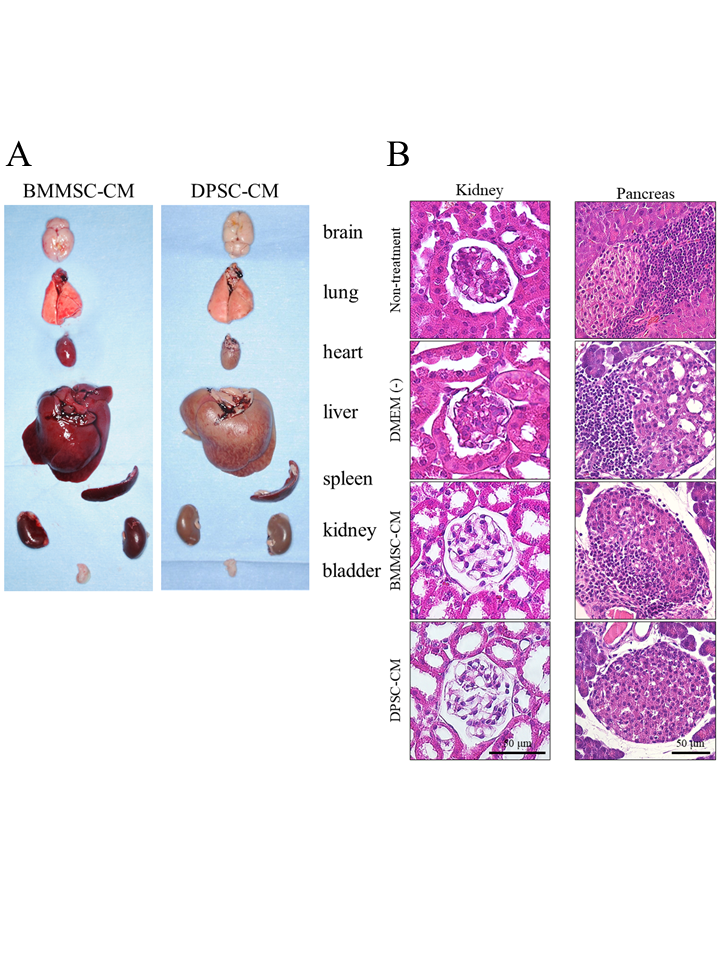
H&E staining was performed as described in the Methods section.

**Supplementary Figure 2.** Evaluation of the safety of BMMSC-CM and DPSC-CM.

(A) Gross images of BMMSC-CM and DPSC-CM injection twice a week for 2 weeks in 15-week-old NOD mice. (B) Representative H&E-stained histological images of the kidney and pancreas of 15-week-old NOD mice. Bars = 50 μm.
