# Supplementary material for: Secreted factors from dental pulp stem cells improve Sjögren’s syndrome via regulatory T cell-mediated immunosuppression
Source: Stem Cell Res Ther. 2021 Mar 16;12:182. doi: 10.1186/s13287-021-02236-6 (PMC7962357; doi:10.1186/s13287-021-02236-6)
Supplement: Supplementary file 3 — Additional file 3: Supplementary Figure 3. Flow cytometric analysis of CD19+IL-10+ and CD20+CD138+ cells. Each graph on the right side shows the percentages of each B cell subset. The data represent the mean ± standard deviation. n = 6. *p < 0.05. [file 13287_2021_2236_MOESM3_ESM.docx]

**Supplementary information**

**Methods**

*Isolation of lymphocytes from the spleen*


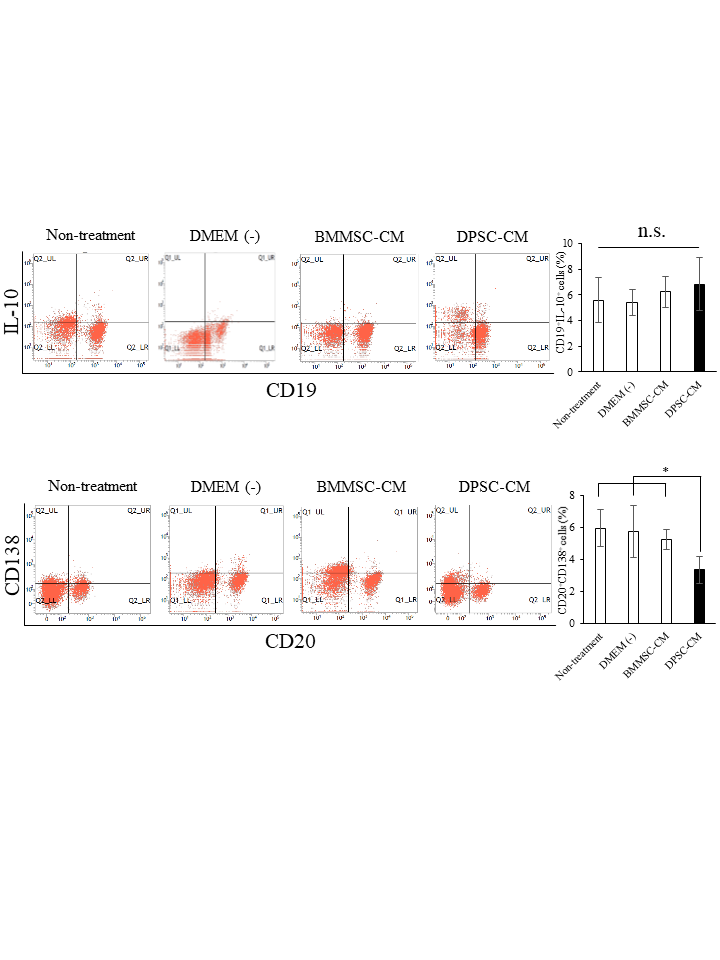
Splenic lymphocyte isolation was performed as described in the Methods section. Lymphocytes were stained with PerCP anti-mouse CD19 (BioLegend), PE anti-mouse IL-10, or PerCP anti-mouse CD20 (BioLegend) and PE anti-mouse CD138 (BioLegend). Thereafter, a BD FACSVerse™ Flow Cytometer (Becton, Dickinson and Company, Franklin Lake, NJ, USA) and BD FACSuite™ software were used to acquire and analyze the FACS data, respectively.

**Supplementary Figure 3.** Flow cytometric analysis of CD19^+^IL-10^+^ and CD20^+^CD138^+^ cells. Each graph on the right side shows the percentages of each B cell subset. The data represent the mean ± standard deviation. *n* = 6. **p* < 0.05.
